# Investigating the Role of MicroRNA396 (miR396) Gene in Regulating Wheat Yield and Grain Nitrogen Concentration

**DOI:** 10.3390/plants15172676

**Published:** 2026-08-31

**Authors:** Yue Yin, Yanwen Yu, Zongliang Ma, Yonghui Wang, Zhiyun Yang, Meng Li, Maosheng Zhu, Danhong Li, Mingyue Gou, Xiaohuan Mu

**Affiliations:** 1 State Key Laboratory of High-Efficiency Production of Wheat-Maize Double Cropping, Collaborative Innovation Center of Henan Grain Crops, Center for Crop Genome Engineering, College of Agronomy, Henan Agricultural University, Zhengzhou 450046, China; 2 The Shennong Laboratory, Zhengzhou 450046, China

**Keywords:** nitrogen, miR396s, TaMIM396, grain yield, grain nitrogen concentration, dry matter, remobilization

## Abstract

Nitrogen (N) is essential for crop growth, yet excessive fertilization causes environmental issues, highlighting the need to sustain yield and grain N concentration under reduced N input. miR396s are known to regulate plant development and stress responses. Here, we examined whether and how miR396 affects wheat yield and N status under high and low N conditions. TaMIM396 (transforming with the target mimicry construct of miR396) overexpression significantly increased plant height, spike length, grain yield, and grain N concentration under both N treatments. Physiological data showed TaMIM396 enhanced dry matter (DM) and N accumulation at anthesis and maturity, as well as improved post-anthesis remobilization of DM and N to grains. RNA-seq analysis revealed that, under low N, TaMIM396 specifically upregulated key photosynthetic antenna genes, including Lhca3 and Lhcb1/2/3/5, which are critical for light harvesting, suggesting improved photosynthetic efficiency that promotes DM accumulation under N limitation. Collectively, our results demonstrate that TaMIM396 acts as a broad-spectrum N-efficiency gene, coordinating carbon and N remobilization while boosting photosynthetic capacity, thereby supporting stable yield and grain N concentration across N supply levels. Therefore, TaMIM396 is a promising candidate for breeding N-efficient wheat cultivars compatible with sustainable high-yield agriculture.

## 1. Introduction

Nitrogen (N) is a critical macronutrient for plants, being a key building block of amino acids, proteins, cell wall components, membranes, and nucleic acids, all of which are fundamental to growth and development [1,2]. Over the last 50 years, crop yield has increased with higher nitrogen fertilizer application [3,4,5,6]. However, excessive N fertilizer application not only triggers serious environmental threats, such as soil acidification, degradation, and water contamination, but also produces potent greenhouse gases (e.g., N_2_O) that exacerbate climate change [7,8]. In this context, achieving high yields under reduced N input presents a major challenge, as lowering N application tends to suppress photosynthesis, restrict leaf expansion, and accelerate senescence, ultimately causing a marked decline in productivity [9]. Therefore, breeding nitrogen-efficient cultivars offers a promising approach to achieving high grain yields under reduced nitrogen input.

Wheat is one of the most important cereal crops grown worldwide. Wheat grain contains high levels of carbohydrates and offers a greater protein content than other major cereals, including rice, maize and millet [3]. As a result, it is commonly used in the production of bread, pasta, and other bakery products. Over the past several decades, wheat grain yield increased with N fertilizer application, while grain N concentration decreased [3,10]. Grain yield is largely determined by dry matter (DM) accumulation after anthesis, which is driven by photosynthesis during the same period [11,12]. Grain N accumulation is derived from post-anthesis N uptake and remobilization of pre-anthesis accumulated N in vegetative organs [12,13]. Improving grain yield and grain nitrogen concentration simultaneously in wheat is an important challenge for breeders.

MicroRNA396 (miR396), as a highly conserved miRNA family in plants, regulates plant growth and development [14,15,16,17] and stress response by targeting growth regulation factors (GRFs) [18,19,20,21]. The modulation of miR396 activity has been shown to affect grain yield in multiple crop species, with effects often manifested under specific conditions [15,16,17,18,19]. The knockout of miR396 in soybean increases seed size and yield, especially under salinity stress conditions [17,19]. In rice, the knockout of miR396ef (but not other isoforms) increased both grain size and panicle branching, thereby boosting grain yield, especially under N deficiency conditions [16,18]. In wheat, TaMIM396 (transforming with the target mimicry construct of miR396) plants showed increased grain size, spike length, and grain number of per spike, and thus improved grain yield [15]. However, despite these observations, whether modulating miR396 expression in wheat can consistently enhance yield and improve nitrogen use efficiency specifically under N-limited conditions remains unclear, as most existing studies have focused on optimal or single-stress environments.

In this study, we investigated the physiological and molecular mechanisms by which miR396 influences wheat grain yield and grain N concentration. We compared wild-type Fielder and TaMIM396 overexpressing lines under both normal and low nitrogen (LN) conditions. Our results demonstrate that TaMIM396 overexpression significantly improves plant height, spike length, and grain yield, primarily by promoting dry matter accumulation and enhancing the remobilization of both biomass and nitrogen. To dissect the mechanistic basis of miR396-conferred tolerance to nitrogen deficiency, we conducted RNA-seq analysis. RNA-seq reveals that TaMIM396 modulates the expression of genes associated with light harvesting and photosynthetic antenna proteins, providing a molecular basis for the observed improvements in carbon and nitrogen metabolism under LN conditions.

## 2. Results

### 2.1. TaMIM396 Positively Regulates Wheat Agronomic Traits and Nitrogen Utilization Under Different Nitrogen Conditions

**Figure 1:**
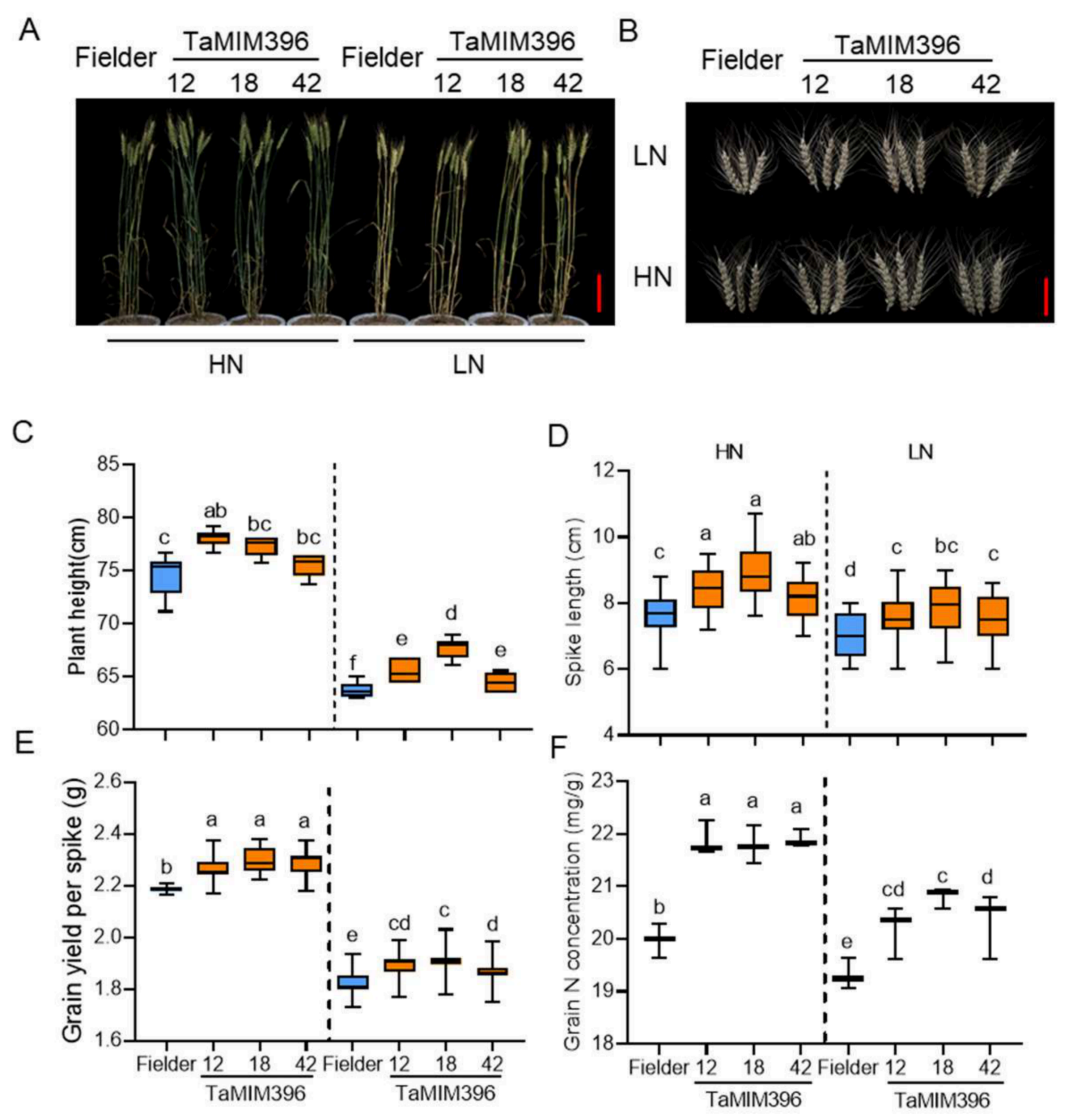
Analysis of agronomic traits of TaMIM396 under different nitrogen treatments. (**A**) Growth traits of Fielder and TaMIM396 lines under high and low nitrogen treatments; bar = 10 cm; (**B**) spike growth traits of Fielder and TaMIM396 lines under high and low nitrogen treatments; bar = 5 cm; (**C**) plant height of Fielder and TaMIM396 lines under high and low nitrogen treatments; (**D**) spike length of Fielder and TaMIM396 lines under high and low nitrogen treatments; (**E**) grain yield per spike of Fielder and TaMIM396 lines under high and low nitrogen treatments; (**F**) grain nitrogen concentration of Fielder and TaMIM396 lines under high and low nitrogen treatments. One-way ANOVA followed by Duncan’s multiple range test was performed by SPSS Statistics 19.0 (SPSS, Inc., Chicago, IL, USA). Different letters above error bars in (**C**–**F**) indicate significant difference at *p* < 0.05.

To investigate the effect of TaMIM396 overexpression on wheat growth and yield under different nitrogen conditions, we grew wild-type Fielder and three independent TaMIM396-overexpressing lines (12, 18, 42) under HN and LN conditions. Plant height and spike length were increased in three TaMIM396 lines compared with Fielder, with the most pronounced differences observed under LN stress (Figure 1A–D). Consistent with previous findings, the grain yield of the TaMIM396 overexpression lines was significantly increased compared with that of the Fielder (Figure 1E) [15], and a similar result was observed under LN stress (Figure 1E). Additionally, grain N concentration was also elevated in TaMIM396 overexpression lines relative to Fielder under both N conditions (Figure 1F).

### 2.2. TaMIM396 Promotes DM Accumulation and Remobilization

Grain yield accumulation was derived from pre-anthesis DM remobilization and post-anthesis DM accumulation [22]. DM accumulations at the anthesis stage and maturity stage of TaMIM396 overexpression lines were significantly increased compared with Fielder under different N treatments (Table 1). Post-anthesis DM accumulation was also substantially enhanced in the TaMIM396 overexpression lines compared with Fielder. What is more, the DM remobilization after anthesis and DM remobilization efficiency were also dramatically increased in TaMIM396 lines compared with Fielder, especially under HN conditions. Consequently, the contribution to grain yield by DM remobilization was higher in three TaMIM396 lines compared with Fielder, especially under the HN condition (Table 1).

### 2.3. TaMIM396 Promotes Nitrogen Uptake and Remobilization

Grain nitrogen accumulation relies on both the post-anthesis N uptake and the N remobilization from vegetative organs [13]. Under both HN and LN conditions, TaMIM396 lines exhibited significantly higher N accumulation than Fielder at both the anthesis and the maturity stage (Table 2). Post-anthesis N accumulation was enhanced in TaMIM396 lines compared with Fielder. N remobilization after anthesis and N remobilization efficiency of TaMIM396 lines were dramatically increased compared with Fielder, especially under HN conditions. Consequently, the contribution of grain N by N remobilization was dramatically increased compared with Fielder.

### 2.4. TaMIM396 Improves Photosynthesis and Thus Increases DW Accumulation Under Low N Stress

**Figure 2:**
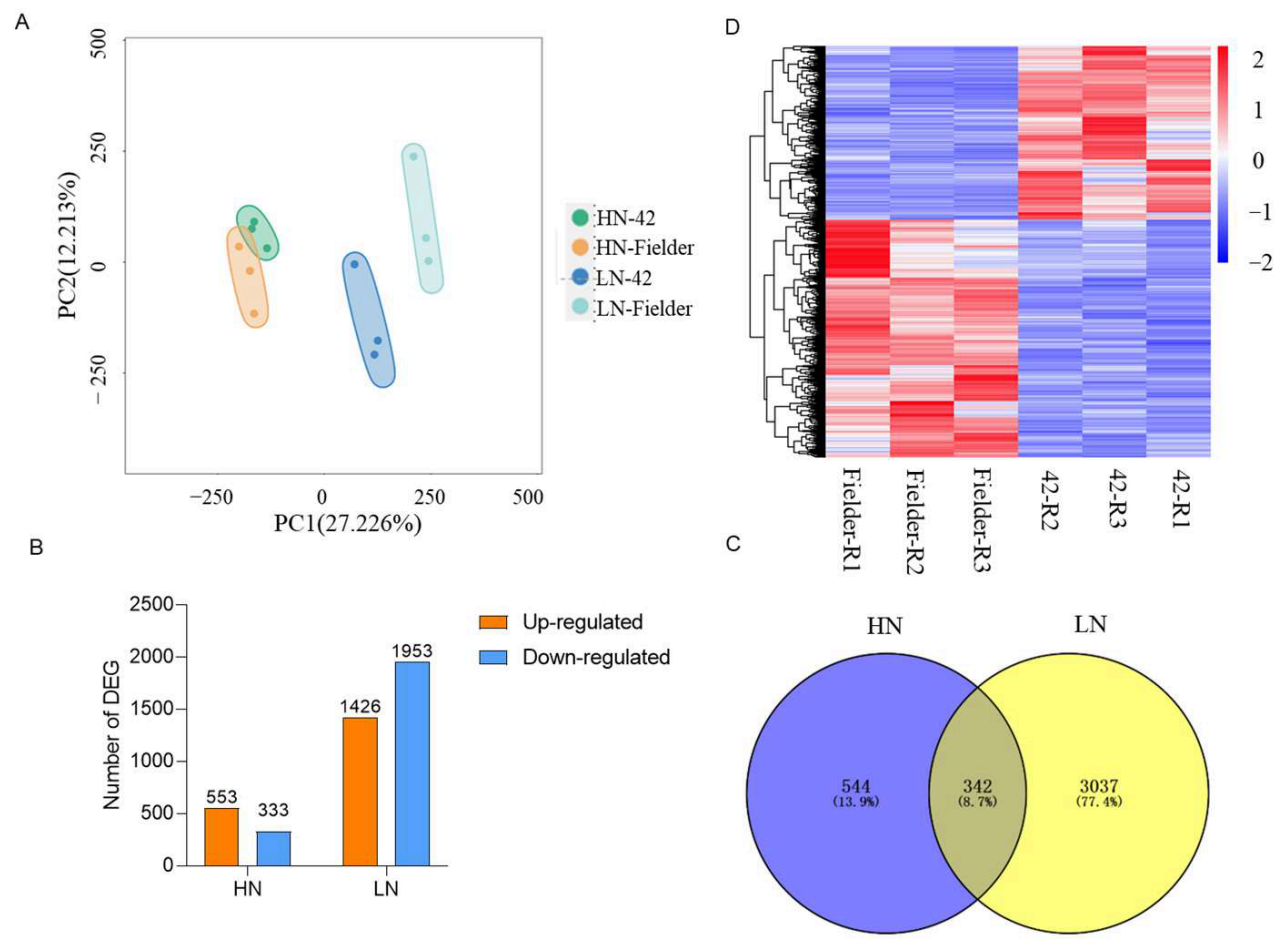
Analysis of differentially expressed genes in the leaves of TaMIM396 versus Fielder under different nitrogen treatments. (**A**) Principal component analysis (PCA) of differentially expressed genes in leaves of Fielder and TaMIM396 line 42 under different nitrogen treatment; (**B**) analysis of the number of differentially expressed genes in leaves of Fielder and TaMIM396 line 42 under different nitrogen treatment; (**C**) Venn diagram displaying DEGs unique and common in TaMIM396 versus Fielder under different nitrogen treatment; (**D**) heatmap clustering analysis of specific DEGs under low nitrogen condition. HN and LN are short for high nitrogen and low nitrogen, respectively.

**Figure 3:**
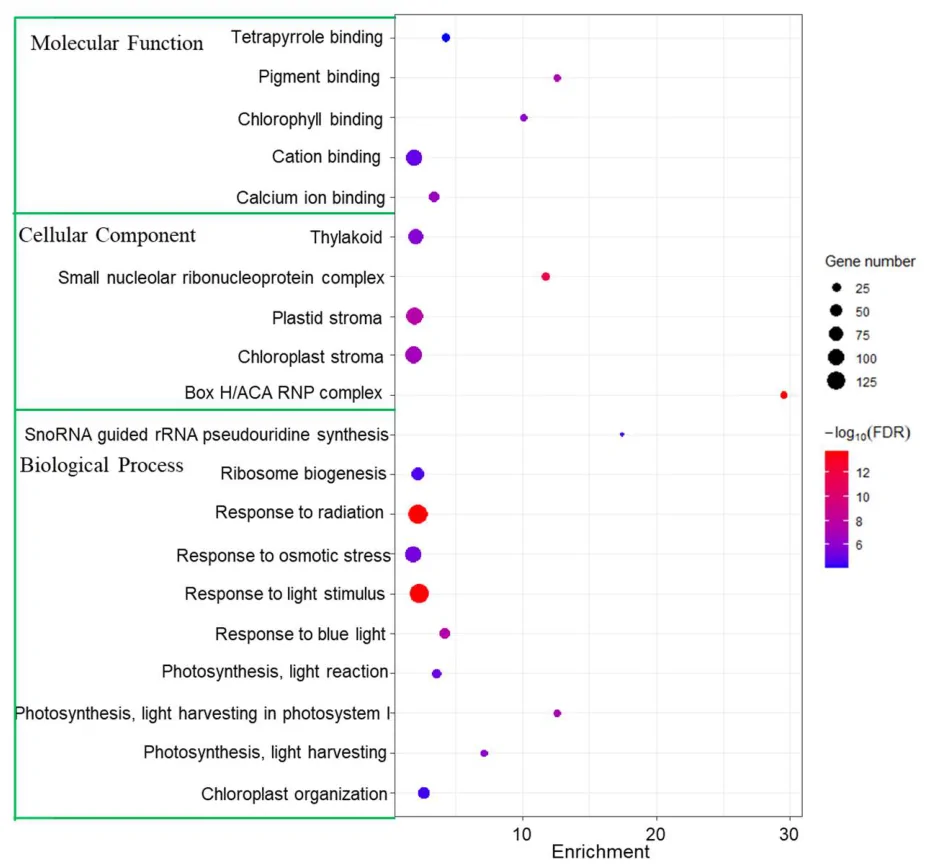
GO enrichment analysis of specific DEGs of TaMIM396 versus Fielder under low nitrogen treatment.

To elucidate the molecular basis underlying miR396-mediated enhancement of tolerance to nitrogen stress, we performed the RNA-seq analysis on leaves of TaMIM396 lines and Fielder at 28 days after N treatment. PCA showed distinct treatment-specific clustering of replicates, indicating good data quality and experimental reproducibility (Figure 2A). Under HN condition, 553 and 333 genes were significantly up- and downregulated in the TaMIM396 line versus Fielder (Figure 2B, Table S1). And there were 1426 upregulated genes and 1953 downregulated genes in the TaMIM396 line versus Fielder under low N treatment (Figure 2B, Table S2). Furthermore, 342 differentially expressed genes (DEGs) were overlapping for HN and LN, and were assigned as common genes (CGs) (Figure 2C, Table S6). There were 3037 LN-specific DEGs and 544 HN-specific DEGs, which were classified as specific genes (SGs) (Figure 2C, Table S3). GO-term enrichment analysis was performed to elucidate the functional enrichment analysis of SGs under LN treatment. There were 254 biological processes (BPs), 31 cellular components (CCs), and 89 molecular functions (MFs) in the GO analysis of SGs under LN treatment (Table S4). SGs under LN treatment were mainly focused on “Response to light stimulus”, “Photosynthesis, light reaction”, “Photosynthesis, light harvesting in photosystem I” and “Photosynthesis, light harvesting” in BP-GO terms; “Thylakoid”, “Plastid stroma” and “Chloroplast stroma” in CC-GO terms; and “Pigment binding”, “Chlorophyll binding” and “Cation binding” in MF-GO terms (Figure 3). All these GO terms have a close relationship with photosynthesis.

**Figure 4:**
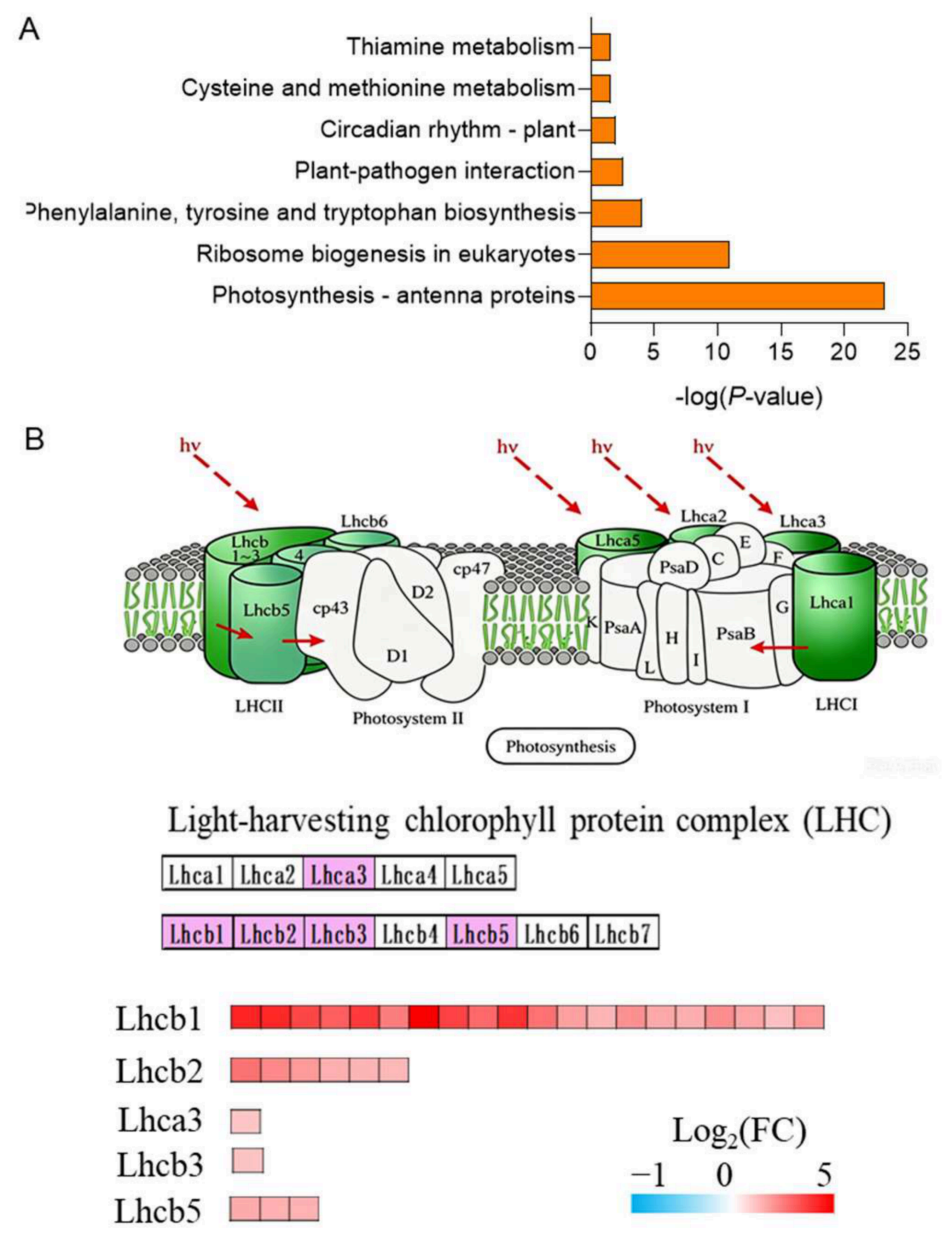
KEGG enrichment and pathway analysis of specific DEGs of TaMIM396 versus Fielder under low nitrogen treatment. (**A**) KEGG enrichment analysis of specific DEGs of TaMIM396; (**B**) analysis of DEGs in the photosynthesis-antenna protein pathway.

KEGG analysis indicated that the SGs of LN were mainly enriched in pathways involved in photosynthesis-antenna proteins, ribosome biogenesis in eukaryotes and phenylalanine, tyrosine and tryptophan biosynthesis and so on (Figure 4A). The DEGs related to photosynthesis-antenna proteins were extracted, and the corresponding pathway map was constructed (Figure 4B). These differentially expressed genes were involved in encoding the photosynthetic light-harvesting pigment protein complex I and light-harvesting pigment protein complex II, mainly including Lhca3, Lhcb1, Lhcb2, Lhcb3, and Lhcb5. Among them, Lhcb1 had the highest number, with 20 members, and all these genes were upregulated in the leaves of TaMIM396 transgenic materials under LN treatment. As light-harvesting antennas, the Lhca and Lhcb family proteins collectively comprise the central apparatus for capturing light energy.

## 3. Discussion

Nitrogen deficiency is a major abiotic constraint on global wheat production, severely limiting vegetative growth, biomass accumulation, and final grain yield [10,23,24]. Consequently, enhancing and/or maintaining grain yield and N concentration under reduced nitrogen fertilizer application has become a primary objective in wheat genetic improvement. In this study, we demonstrate that the overexpression of TaMIM396 substantially promotes growth, grain yield, and grain N concentration in wheat, particularly under nitrogen-deprived conditions. Beyond confirming the positive regulatory function of TaMIM396 in key agronomic traits, our work provides mechanistic insights into the physiological and molecular basis of this enhancement, positioning TaMIM396 as a promising candidate gene for breeding nitrogen-efficient wheat cultivars.

### 3.1. TaMIM396 Enhances Yield and Nitrogen Concentration Under High and Low Nitrogen Stress

Our phenotypic analyses revealed that TaMIM396-overexpressing lines exhibited increased plant height, spike length, and grain yield compared with the wild-type Fielder under both N treatments (Figure 1A–F). These results are consistent with previous reports showing that the mutation of Gm-miR396 enhances seed production under salinity stress [17,19], and that the loss of function of MIR396e and MIR396f boosts grain yield in rice under nitrogen limitation [18]. Collectively, our findings indicate that TaMIM396 is particularly effective in mitigating the adverse effects of nitrogen deficiency, a critical feature for sustainable agriculture in low-input systems. Importantly, the concomitant increase in grain nitrogen concentration in the transgenic lines indicates that the improved yield is not achieved at the expense of grain protein content, a common trade-off in many high-yielding cultivars (Figure 1D). This dual benefit—enhanced yield and grain N content—positions TaMIM396 as a valuable genetic resource for simultaneously addressing food security and nutritional quality.

### 3.2. Physiological Basis: Promoted Dry Weight and Nitrogen Accumulation and Remobilization

Grain yield and nitrogen concentration in cereal crops are intrinsically linked to the accumulation and subsequent efficient remobilization of both dry matter and nitrogen throughout the growth cycle [11,12,25]. To dissect the basis of increased grain yield and nitrogen concentration, we examined DM and N accumulation at anthesis and the maturity stage. Our data demonstrated that TaMIM396 overexpression significantly elevated DM accumulation at anthesis and maturity stages, as well as post-anthesis DM remobilization capacity and remobilization efficiency, especially under HN treatment, leading to a greater contribution of remobilized DM to grain yield (Table 1). Mechanistically, the upregulation of fructan biosynthetic genes, specifically those encoding sucrose: sucrose 1-fructosyltransferase-like and sucrose: fructan 6-fructosyltransferase, in TaMIM396 lines relative to Fielder under HN treatment (Table S7) may underpin this improved remobilization. By contrast, under low nitrogen (LN) conditions, the overexpression lines exhibited an upregulated expression of genes encoding sugar and sucrose transporters compared with Fielder (Table S8). Collectively, these findings suggest that TaMIM396 optimizes the source–sink relationship by promoting both the accumulation of vegetative reserves before anthesis and their efficient translocation to developing grains after anthesis. This pattern is agronomically advantageous, as the efficient remobilization of stored carbohydrates to the developing grain ensures stable yield under stress conditions, particularly when post-anthesis photosynthesis may be constrained [12].

Similarly, total N accumulation at flowering and maturity was higher in TaMIM396 lines and thus good for N remobilization from vegetative organs to the grain (Table 2). The increased N remobilization efficiency and its greater contribution to grain N suggest that TaMIM396 can effectively promote nitrogen remobilization to grains, which is conducive to the simultaneous improvement of wheat yield and grain quality under both sufficient and deficient nitrogen supply (Table 2). Collectively, these physiological data indicate that TaMIM396 coordinates the mobilization of both carbon and nitrogen resources, optimizing their allocation to reproductive structures.

### 3.3. Molecular Mechanism of miR396-Mediated Enhancement of Tolerance to Nitrogen Stress: TaMIM396 Upregulates Photosynthetic Machinery

DM accumulation has a close relationship with photosynthesis, which is severely impaired by nitrogen stress [23,24]. To uncover the molecular basis of enhanced DM and N accumulation under LN stress, we performed comparative transcriptomic analysis. A large number of nitrogen-specific DEGs were identified, including 3037 LN-specific DEGs and 544 HN-specific DEGs, with only a small proportion of overlapping DEGs, indicating that TaMIM396 regulates distinct transcriptional networks in response to different nitrogen environments (Figure 2C). To elucidate the molecular basis underlying the miR396-mediated enhancement of tolerance to nitrogen stress, we analyzed the LN-specific DEGs. GO enrichment analysis of LN-specific DEGs revealed significant enrichment in photosynthesis-related biological processes, cellular components, and molecular functions, including light stimulus response, photosynthetic light reaction, photosystem I light harvesting, thylakoid and chloroplast stroma components, and chlorophyll and pigment binding activities (Figure 3). These results strongly suggest that TaMIM396 mediates wheat low nitrogen tolerance mainly by modulating photosynthetic machinery function. Further KEGG pathway analysis confirmed that LN-specific DEGs were predominantly enriched in photosynthesis-antenna protein pathways, ribosome biogenesis, and amino acid biosynthesis pathways, which are closely associated with light energy capture, protein synthesis, and plant stress adaptation (Figure 4A). What is more, core genes encoding photosynthetic light-harvesting complexes, including Lhca3 and Lhcb1/2/3/5, were significantly upregulated in TaMIM396 lines under low nitrogen conditions (Figure 4B). As key components of photosystem I and II, LHCA and LHCB family proteins constitute the core light-harvesting system of plants [25]. The massive upregulation of these genes effectively maintains the integrity and stability of photosynthetic antenna systems under nitrogen deficiency, improves light energy capture, and ultimately promotes dry matter accumulation and sustainable plant growth.

### 3.4. TaMIM396 Coordinates Carbon and Nitrogen Metabolism to Enhance Yield and Nitrogen Use Efficiency

TaMIM396 overexpression promotes overall plant growth and biomass accumulation, as evidenced by increased plant height, spike length, grain yield, and dry matter accumulation under both high and low nitrogen conditions (Figure 1; Table 1). Larger plants with greater biomass inherently require more nitrogen to support their expanded photosynthetic tissues, protein synthesis, and metabolic activities. Thus, the improved NUE observed in TaMIM396 lines can be attributed, at least in part, to a biomass-driven nitrogen demand: enhanced carbon assimilation and growth create a stronger sink for nitrogen, which in turn promotes nitrogen uptake and remobilization from vegetative organs to developing grains.

At the molecular level, our transcriptomic data revealed that TaMIM396 significantly upregulates photosynthetic light harvesting genes (*Lhca3* and *Lhcb1/2/3/5*) under low nitrogen stress, which enhances photosynthetic efficiency and drives carbon assimilation. The increased carbon supply provides energy and carbon skeletons for nitrogen assimilation, while the larger plant sink promotes nitrogen uptake and remobilization. This coordinated regulation of carbon and nitrogen metabolism ultimately supports higher grain yield and grain nitrogen concentration.

Taken together, TaMIM396 coordinates carbon and nitrogen remobilization and enhances photosynthesis, promoting wheat growth, stable yield, and grain N concentration under both high and low N conditions, making it a promising target for breeding N-efficient cultivars. Mechanistically, TaMIM396 upregulates photosynthetic light-harvesting genes under N limitation, improving light capture and pre-anthesis DM accumulation for subsequent remobilization to grains. Concurrently, it enhances N uptake and, more importantly, N remobilization from senescing tissues to support grain filling. The coordinated increase in both carbon and N fluxes to the grain underlies the observed improvements in yield and grain N content.

## 4. Materials and Methods

### 4.1. Plant Materials, Growth Condition, and Sampling

To investigate the effect of miR396 on wheat growth, TaMIM396 overexpression lines (12, 18 and 42) [15] and the wild-type control (Fielder) were grown in the experimental field at Zhengzhou, China. TaMIM396 lines exhibited significantly reduced miR396 expression compared with wild-type controls [15]. There were two N application levels, 180 kg N ha^−1^ (HN) and zero N (LN) treatment. In the HN treatment, 120 kg N ha^−1^ was supplied as the basal fertilizer and the remaining as the side-dressing at the jointing stage. In the LN treatment, no N fertilizer was applied throughout the entire growing season. The experiment was arranged in a randomized complete block design with four replicates per treatment. Plots were maintained free of weeds, pests, and diseases.

At the anthesis stage and the maturity stage, 6–8 individual plants per plot were cut at soil surface and divided into stems (including glumes and rachises), leaves, and grains. Then the samples were dried to a constant weight at 65 °C and weighed and ground to powder. Subsequently, N concentration was determined using the semi-micro-Kjeldhal method.

### 4.2. Plant Hydroponic Culture and Sampling

Seeds of TaMIM396 lines (12, 18, and 42) and Fielder were sterilized in 10% H_2_O_2_ for 30 min and washed with distilled water. Then, the seeds were placed on a seed starter tray. An appropriate amount of distilled water was added to the seed starter tray, and the water level just slightly covered the bottom of the wheat seeds. Then, they were cultivated in a greenhouse at 25 °C with a 14 h photoperiod. The seedlings with two visible leaves were then transferred into pots (20 seedlings per pot) containing 8 L of nutrient solution. Every plot cultivated five plants of TaMIM396 lines and Fielder.

The nutrient solution contained 1.5 mM KCl, 0.2 mM KH_2_PO_4_, 1.0 mM MgSO_4_, 0.1 mM EDTA-Fe, 1.0 μM MnSO_4_, 1.0 μM ZnSO_4_, 0.05 μM CuSO_4_, 0.005 μM (NH_4_)_6_Mo_7_O_24_ and 10 μM H_3_BO_3_ [1]. Plants were first grown for two days in a half-strength nutrient solution containing 2.0 mM NO_3_^−^ (supplied as Ca(NO_3_)_2_·4H_2_O). They were then transferred to solutions containing either 0.1 mM NO_3_^−^ (LN treatment) or 2.0 mM NO_3_^−^ (HN treatment), with CaCl_2_ added to the low nitrogen solution to balance calcium concentrations between the two groups. The pH of the solution was adjusted to 5.8–6.0 with KOH. The solution was replaced every 4 days and continuously aerated using a pump.

### 4.3. RNA-Seq and Data Analysis

To clarify the physiological and molecular mechanisms driving the differences in dry matter and nitrogen accumulation between the TaMIM396 line and the Fielder cultivar, leaf sampling took place at 28 days of hydroponic culture. Three replicates were collected, each containing five plants. Total RNA was isolated using Trizol reagent and then sequenced on an Illumina NovaSeq platform (Illumina, Inc., San Diego, CA, USA). Clean data were aligned to wheat IWGSC_v2.1 using HISAT2 [26]. Alignments were processed using StringTie software to assemble transcript isoforms and quantify expression levels in fragments per kilobase of exon model per million mapped reads (FPKM) for both known and novel genes [27]. The R package edgeR7 was used to detect differentially expressed genes (DEGs) with fold change >2 and false discovery rate (FDR) less than 0.05 [28]. Venn diagrams were generated using Venny 2.1 (http://bioinfogp.cnb.csic.es/tools/venny/index.html) (accessed on 3 May 2024). Gene ontology (GO) analysis was performed with AgriGO_v2.0 (http://systemsbiology.cau.edu.cn/agriGOv2/) (accessed on 20 August 2024). Kyoto Encyclopedia of Genes and Genomes (KEGG) was identified by R packages clusterProfiler v4.2.

### 4.4. Calculations

Based on the measurements described above, the following parameters were calculated [29].

Vegetative DM (N) remobilization efficiency (%) = 100 × [vegetative DM (N content) at anthesis vegetative DM (N content) at maturity]/vegetative DM (N content) at anthesis;

Contribution to grain yield (N) by vegetative DM (N) remobilization (%) = 100 × [vegetative DM (N content) at anthesis vegetative DM (N content) at maturity]/grain yield (N content) at maturity.

### 4.5. Statistical Analysis

One-way ANOVA followed by Duncan’s multiple range test were performed by SPSS Statistics 19.0 (SPSS, Inc., Chicago, IL, USA). Differences were compared by least difference test at *p* < 0.05.

## Figures and Tables

**Table 1:** Effect of TaMIM396 on pre- and post-anthesis dry matter (DM) accumulation, remobilization and contribution to grain yield.

Treatment	Genotype		DM Accumulation at Anthesis Stage	DM Accumulation at Maturity Stage	DM Remobilization After Anthesis	DM Remobilization Efficiency	**Contribution to Grain Yield by DM Remobilization**
			(g/Spike)	(g/Spike)	(g/Spike)	(%)	**(%)**
HN	Fielder		2.52 c	4.13 b	0.58 b	22.98 c	26.52 cd
	TaMIM396	12	2.72 ab	4.26 a	0.71 a	26.28 b	31.46 bc
		18	2.77 b	4.27 a	0.84 a	29.67 ab	36.73 ab
		42	2.95 a	4.30 a	0.93 a	31.69 a	40.79 a
LN	Fielder		1.90 f	3.41 d	0.31 d	16.10 de	16.91 e
	TaMIM396	12	2.14 d	3.54 c	0.48 c	22.69 cd	25.66 cd
		18	2.08 de	3.58 c	0.41 cd	19.69 d	21.69 de
		42	1.95 ef	3.54 c	0.28 d	14.29 e	15.02 e

Note: HN and LN are short for high nitrogen and low nitrogen, respectively. One-way ANOVA followed by Duncan’s multiple range test were performed by SPSS Statistics 19.0 (SPSS, Inc., Chicago, IL, USA). Different letters indicate in one column significant differences (*p* < 0.05).

**Table 2:** Effect of TaMIM396 on pre- and post-anthesis nitrogen (N) accumulation, remobilization and contribution to grain yield.

Treatment	Genotype		N Accumulation at Flowering Stage	N Accumulation at Maturity Stage	N Remobilization After Anthesis	N Remobilization Efficiency	Contribution to Grain N by N Remobilization
			(g/Spike)	(g/Spike)	(g/Spike)	(%)	(%)
HN	Fielder		43.78 b	59.36 b	28.06 b	63.98 e	64.39 bc
	TaMIM396	12	50.65 a	64.77 a	35.46 a	69.98 cd	71.55 ab
		18	52.23 a	65.50 a	35.92 a	68.68 d	71.87 ab
		42	54.67 a	66.32 a	39.29 a	71.78 bcd	78.37 a
LN	Fielder		25.61 d	42.53 d	18.32 d	71.40 bcd	52.08 d
	TaMIM396	12	30.02 c	45.94 c	22.27 cd	74.14 ab	58.39 cd
		18	31.99 c	46.66 c	24.28 bc	75.82 a	61.21 bcd
		42	33.12 c	47.40 c	24.37 bc	73.56 abc	64.27 bc

Note: HN and LN are short for high nitrogen and low nitrogen, respectively. One-way ANOVA followed by Duncan’s multiple range test were performed by SPSS Statistics 19.0 (SPSS, Inc., Chicago, IL, USA). Different letters indicate in one column significant differences (*p* < 0.05).

## Data Availability

Data of RNA-Seq in this study have been deposited in the NCBI Sequence Read Archive (SRA) database (accession no. PRJNA1501651).

## Supplementary Materials

The following supporting information can be downloaded at: https://www.mdpi.com/article/10.3390/plants15172676/s1, Table S1: The differentially expressed genes inTaMIM396-overexpressing line versus Fielder under HN treatment. Table S2: The differentially expressed genes inTaMIM396-overexpressing line versus Fielder under LN treatment. Table S3: The specific-DEGs of TaMIM396-overexpressing line versus Fielder under LN treatment. Table S4: Gene ontology term analysis of specific-DEGs in TaMIM396-overexpressing line versus Fielder under LN treatment. Table S5: Kyoto Encyclopedia of Genes and Genomes analysis of specific-DEGs in TaMIM396-overexpressing line versus Fielder under LN treatment. Table S6: The common differentially expressed genes of TaMIM396 versus Fielder under different N treatment. Table S7: The genes involved in fructan biosynthesis in TaMIM396 versus Fielder under HN treatment. Table S8: The genes involved in sugar and sucrose transporter in TaMIM396 versus Fielder under LN treatment.
